# Clinical and Genetic Interplay Between Chronic Pancreatitis, Cystic Fibrosis and CFTR-Related Disorders

**DOI:** 10.3390/ijms27198620

**Published:** 2026-09-26

**Authors:** Carnovale Vincenzo, Tosco Antonella, Castaldo Alice, Fevola Cristina, Comegna Marika, Gelzo Monica, Iacotucci Paola, Sepe Angela, Terlizzi Vito, Giuseppe Castaldo

**Affiliations:** 1 Centro di Riferimento Regionale Campano CF Adulti, Dipartimento di Scienze Mediche Traslazionali, Università di Napoli Federico II, 80131 Naples, Italy; vincenzo.carnovale@unina.it (C.V.); paola.iacotucci@unina.it (I.P.); 2 Centro di Riferimento Regionale per La Cura della Fibrosi Cistica Pediatrico, Dipartimento Materno-Infantile, Azienda Ospedaliera Universitaria Federico II, 80131 Naples, Italy; antonellatosco@gmail.com (T.A.); ornellasepe@hotmail.com (S.A.); 3 CEINGE-Biotecnologie Avanzate Franco Salvatore, 80131 Naples, Italy; alicastaldo94@gmail.com (C.A.); monica.gelzo@unina.it (G.M.); giuseppe.castaldo@unina.it (G.C.); 4 SC di Pneumologia e UTSIR, AORN Santobono-Pausilipon, 80129 Naples, Italy; 5 Centro di Riferimento Regionale Toscano sulla Fibrosi Cistica, Azienda Ospedaliera Universitaria Meyer IRCCS, 50139 Florence, Italy; cristina.fevola@meyer.it (F.C.); vito.terlizzi@meyer.it (T.V.); 6 Centro di Riferimento Regionale Campano CF, Dipartimento di Medicina Molecolare e Biotecnologie Mediche, Università di Napoli Federico II, 80131 Naples, Italy; 7 Dipartimento di Scienze Biomediche Avanzate, Università di Napoli Federico II, 80131 Naples, Italy

**Keywords:** idiopathic chronic pancreatitis, hereditary pancreatitis, oligogenic inheritance, trans-heterozygosity, genotype–phenotype correlation, genetic testing, precision medicine

## Abstract

Chronic pancreatitis (CP) is a clinically heterogeneous condition that may occur with a broad clinical spectrum, including cases of cystic fibrosis (CF), cystic fibrosis transmembrane regulator (CFTR)-related disorder (CFTR-RD), acute recurrent pancreatitis (ARP), and CP, including cases of idiopathic CP (iCP) and hereditary pancreatitis (HP). The specific contribution of *CFTR* variants and pancreatitis-predisposing genes (PPG), i.e., genes responsible for intrapancreatic activation of trypsinogen or pancreatic secretion pathways to CP susceptibility in these different contexts remains incompletely understood. We investigated the occurrence of CP and mutations in PPG genes in: (i) 377 subjects with CF and (ii) 217 subjects with CFTR-RD (including cases expressing with CP alone). Moreover, we studied 136 subjects with iCP. CP occurred in 33/377 subjects (8.8%) with CF carrying *CFTR* genotypes other than homozygous p.Phe508del and was mainly associated with pancreatic sufficiency (24/33, 72.7%) and at least one residual-function *CFTR* variant (25/33, 75.8%). Chronic pancreatitis also occurred in 8 CF subjects carrying both *CFTR* mutations classified as no residual function, and 7 of them also had PPG variants. Subjects with CFTR-RD expressing with ARP/CP alone (*n* = 92) are genetically indistinguishable from CFTR-RD subjects with diffuse bronchiectasis (*n* = 45) or congenital bilateral absence of vas deferens (*n* = 92). In fact, all CFTR-RD subjects had at least one residual-function *CFTR* variant, all had pancreatic sufficiency, and the sweat chloride was not significantly different between the three subgroups. Finally, 66/136 (48.5%) subjects with iCP harbored pathogenic variants in PPG, with 26/66 cases (i.e., 39.4%) with multiple gene mutations (consistent with a potential role of oligogenic inheritance in CP susceptibility). Idiopathic CP subjects with mutations in PPG (48.5% in our series) are known to be at higher risk to develop pancreatic adenocarcinoma. Our study revealed such mutations also in subjects with CF (with both *CFTR* mutations classified as no residual function and with pancreatic insufficiency) and in subjects with CFTR-RD, supporting the use of multigene testing and genetic counselling. To conclude: The early diagnosis of CF by NBS enhanced the number of CF subjects who develop ARP and/or CP, independently by the pancreatic status and by the *CFTR* genotype. Mutations in PGP genes further increase such risk. Thus, we suggest: (i) to test for PGP genes and counseling all cases of CF with pancreatitis; (ii) large multicenter studies to investigate why a subset of CFTR-RD appear with ARP/CP alone; and (iii) a careful investigation of familiarity and the analysis of a large panel of PPG genes to better define the profile of risk for HP in all cases of idiopathic ARP/CP.

## 1. Introduction

Chronic pancreatitis (CP) is a persistent inflammation of the pancreas with ductal abnormalities that gradually lead to fibrosis and pancreatic atrophy, compromising exocrine and endocrine functions. The annual incidence of 5 to 8 and prevalence of 42 to 73 cases per 100,000 adults have been reported in the United States. Higher prevalence rates were reported in India and in other Asian ethnic groups. The male-to-female ratio is approximately 2:1. Gallstones, drugs, and hypertriglyceridemia are known etiologic factors for CP alcohol abuse, smoking, autoimmunity, and hypercalcemia, even in combination, are the most common risk factors in adults [1], while in children a significant percentage of CP is due to congenital pancreatic malformations [2]. Approximately one-third of cases are idiopathic. Among these, hereditary pancreatitis (HP) appears in the pediatric age or in young adults with acute, acute recurrent (ARP) or with CP in the absence of known etiological factors, often with other affected family members and mutations in predisposing genes [1,2]. In recent years, HP has attracted great interest due to the enhanced risk of ductal adenocarcinoma [3].

The relationship between CP and ARP remains unclear. Acute recurrent pancreatitis is characterized by at least two well-documented episodes of acute pancreatitis, separated by a period of complete clinical resolution. Acute recurrent pancreatitis is reversible once the etiological factors are removed, while CP is considered a non-reversible condition [4], even if most authors consider ARP as a precursor of CP [3]. Among the genes related to the pathogenesis of CP, some have a direct autosomal dominant effect, and others act as modifiers of the pancreatic phenotype, potentiating the effect of other genes [5]. We previously studied these genes both in subjects affected by cystic fibrosis (CF) and CP and in subjects with CP not affected by CF [6,7], hypothesizing trans-heterozygosity as a novel mechanism of inheritance for CP. These genes may be defined as pancreatitis-predisposing genes (PPG) and are involved in the intrapancreatic activation of trypsinogen (IPAT) or in the pancreatic secretion pathways (PSP).

Adding to the complexity of such an intricate issue is the evidence indicating that individuals with cystic fibrosis are at increased risk of developing ARP or CP [8]. In fact, CF subjects carrying both *CFTR* mutations with no residual function have more viscous pancreatic secretions, creating obstructive tubular damage and subsequent intrapancreatic activation of pancreatic enzymes, auto-digestion, and fibrosis, causing the replacement of the exocrine tissue and generating pancreatic insufficiency (PI), and the absence of residual exocrine tissue prevents the development of pancreatitis [8]. While, in CF subjects that bear at least one mutation with residual *CFTR* function, such latter exerts a dominant effect on the pancreatic status and warrants a residual activity of the exocrine tissue at the price of a higher risk to develop acute recurrent and/or CP [8]. The newborn screening (NBS) enhanced the number of CF-diagnosed individuals carrying the mutation with residual *CFTR* function, thus causing an increase in CF cases that will potentially develop pancreatitis. Moreover, the treatment with modulators of the *cystic fibrosis transmembrane conductance regulator* (*CFTR*) gene restores some functionality of the pancreatic acinar tissue, causing on the turn an enhanced risk of developing pancreatitis. Finally, some *CFTR* mutations directly impacting the development of ARP and CP in the absence of CF were identified [9]. Furthermore, among subjects with CFTR-related disorders (CFTR-RD), often characterized by at least one *CFTR* variant with residual CFTR function [10] and a monosymptomatic clinical picture, a group of subjects suffer from isolated ARP or CP.

Therefore, to evaluate the relationships between CP, CF, CFTR-RD, and variants in *CFTR* and PPG, we studied: (i) 377 adult or pediatric subjects with CF and different *CFTR* genotypes; (ii) 217 subjects with CFTR-RD (with the three main clinical phenotypes, namely CP, diffuse bronchiectasis (DB), and congenital bilateral absence of the vas deferens (CBAVD); and (iii) 136 subjects with idiopathic CP (iCP).

## 2. Results

### 2.1. Adult and Pediatric CF Subjects Homozygous for the p.Phe508del Mutation Have a More Severe Disease as Compared to CF Subjects with Other Genotypes, and No Risk to Develop Chronic Pancreatitis

Table 1 compares the values of sweat chloride (SC), the pancreatic status, and the number of cases with CP between CF subjects homozygous for the p.Phe508del mutation and those with other *CFTR* genotypes in adult and pediatric subgroups. In both the subgroups, the values of SC resulted significantly higher in subjects homozygous for the p.Phe508del mutation as compared to those with other genotypes (*p* < 0.001). Moreover, all (but one) adult and all pediatric CF subjects homozygous for the p.Phe508del had PI, while among subjects with other *CFTR* genotypes we recorded 69/193 (35.8%) and 22/69 (31.9%) adult and pediatric cases, respectively, with PS. Finally, cases of CP (i.e., 21 cases in the adult and 12 in the pediatric subgroup) were recorded only among subjects without the p.Phe508del homozygous genotype (other genotypes).

### 2.2. Adult and Pediatric CF Subjects with CP Have a Less Severe Disease and a Higher Percentage of CFTR Mutations Associated with Residual Function as Compared to CF Subjects Free from CP

Table 2 reports the main features of the 21 adult and of the 12 pediatric CF subjects with CP as compared to adult and pediatric subgroups of CF subjects free from CP, respectively. In both the subgroups, the values of SC resulted significantly lower in CF subjects with CP as compared to CF subjects free from CP (*p* < 0.006 for adult and *p* < 0.001 for pediatric subjects). Moreover, the number of cases with PI was significantly lower among adult CF subjects with CP (*p* < 0.001), while the opposite result was obtained for the number of cases with PS, and this was true also for pediatric CF subjects (*p* < 0.001). For each CF subject we considered the *CFTR* mutation class (i.e., class 1–3 for mutations with no residual activity and class 4–6 for mutations with some residual activity). Thus, we compared the number of CF subjects with no residual function *CFTR* mutations (i.e., class 1–3) with CP with those free from CP either in the adult or in the pediatric subgroup of CF. In both the subgroups, the number of cases with two class 1–3 mutations was significantly lower among CF subjects with CP as compared to CF subjects free from CP (*p* < 0.001, for both adult and pediatric subjects). The opposite result was obtained comparing the number of cases with a class 1–3 and a residual function (i.e., class 4–6) mutation.

Supplemental Table S1 reports the *CFTR* genotypes found in all 33 CF subjects with CP; 25 subjects had a compound heterozygous genotype between a no-residual function (i.e., class 1–3) and a residual function (i.e., class 4–6) *CFTR* mutation, and 8/33 (24.2%) had two class 1–3 *CFTR* mutations. Supplemental Table S2 shows all the mutations found in CF subjects with CP in comparison with CF subjects free from CP. The frequency of the p.Phe508del resulted significantly higher among CF subjects free from CP, while the frequency of other mutations, i.e., p.Asp1152His, c.2789 + 5G > A, p.Leu997Phe, and p.Gly85Glu resulted in significantly higher CF subjects with CP.

Moreover, Supplemental Table S1 reports the mutations in genes bearing the intra-pancreatic activation of the trypsinogen pathway (IPAT genes) found in the 33 subjects with CP. Eight subjects had an IPAT gene mutation. Of these, seven had two class 1–3 mutations, while one had a class 1–3 and a class 4–6 *CFTR* mutation. Finally, Supplemental Table S3 reports the number of mutations in IPAT genes found in the 8 CF subjects with CP in comparison with CF subjects free from CP. Only 5/344 (1.5%) CF subjects free from CP had a mutation in an IPAT gene, a figure significantly lower (*p* < 0.001) as compared to the 8/33 (24.2%) cases with an IPAT gene mutation found in subjects with CP.

### 2.3. Subjects with CFTR-RD That Appear with CP Are Not Distinguishable from CFTR-RD Subjects That Appear with DB or CBAVD

Table 3 shows the main features of 217 subjects with CFTR-RD and a monosymptomatic clinical expression, i.e., 80 with CP, 45 with DB, and 92 with CBAVD. The median values of SC are 36, 39, and 33 mEq/L, respectively, and none of the 217 subjects had SC >60 mEq/L. Moreover, all the subjects had PS, and all but 4 had a *CFTR* genotype that included a class 1–3 and a class 4–6 mutation. Thus, no significant differences were recorded for the above-mentioned parameters among the three subgroups of subjects with CFTR-RD. Supplemental Table S4 lists the number and % of *CFTR* mutations found in the three subgroups of subjects. Only p.Asp1152His resulted in significantly more frequent (*p* < 0.010) subjects with CP (i.e., the p.Asp1152His, the p.Leu997Phe), while no differences were recorded for all the other mutations. Finally, we analyzed the genes of the IPAT pathway in all 217 subjects. No mutations were found in any of the examined cases.

### 2.4. About Half of Subjects with Idiopathic CP Display Mutations in Genes of the IPAT Pathway (The Highest Number Involves CFTR) and of the PSP

We studied 136 subjects with idiopathic chronic pancreatitis. Of these, 44 (32.4%) had iARP not still evolved to iCP, and the remaining 92 had CP, but all subjects in the present study are defined as iCP according to the more current view that ARP is a precursor of CP (3). The median age at diagnosis was 27 years, with 58 cases (42.7%) diagnosed in the pediatric age. There was a slightly higher number of males (78/136, 57.3%). Family history was observed in 36 cases (26.5%). As shown in Supplemental Table S5, 66/136 (48.5%) cases had one or more mutations in the IPAT pathway or in PSP genes. Among the 66 subjects with mutations, 4 had mutations in three different genes, 22 had mutations in two different genes, and 40 had one mutation in a single gene. Supplemental Table S6 lists the mutations of genes related to pancreatitis in the 66 subjects with CP. Among the mutations of the *IPAT* genes, 28 alleles had one of 13 *CFTR* mutations, 14 alleles had one of 6 *PRSS1* mutations, 8 alleles had one of 2 *SPINK1* mutations, 3 had one of 2 *PRSS2* mutations,, 6 had one of 4 *CTRC* mutations and one had a mutation of the *KRT8* or of the *CTSB* gene. While, among the mutations in genes encoding proteins of the PSP pathway, 4 different mutations of the *SERPINA1* gene were found in six alleles, 3 distinct mutations were found in single alleles for *TMPRSS15* and *CPA1* genes, 2 different mutations were identified in single alleles for *CEL* and *SLC26A3* genes, and finally, a single mutant allele was found for *ATP2C2*, *DMBT1*, *KMT2D*, *IL21*, *PNILP*, *SLC4A4*, *STIM1*, *TNFRSF1A*, *TRPV1,* and *TRPV5* genes.

## 3. Discussion

We firstly explored the relationships between CF and CP, evaluating 377 adult and pediatric CF subjects homozygous for the p.Phe508del mutation or with other *CFTR* genotypes. We found 33/377 cases of CP (8.8%), of which 24 were among the 92 CF subjects with PS (26.1%) and 9 were among the 285 CF subjects with PI (3.1%). Such figures are higher as compared to 0.9% of CF subjects with PI and to 2.9% of CF subjects with PI who develop pancreatitis [11] and to the overall frequency of about 20% of CF subjects with PS that develop CP during the course of life [8]. Likely, it depends on the higher number of CF subjects with PS in our series since all subjects were diagnosed by the NBS. In fact, in our study we found 92/377 CF subjects (24.4%) with PS, compared to the figure of about 15% of CF subjects with PS commonly reported [8].

Interestingly, no cases of CP were found among CF subjects with the p.Phe508del homozygous genotype. The p.Phe508del, as well as other severe *CFTR* mutations, causes more viscous pancreatic secretions, creating obstructive tubular damage and subsequent intrapancreatic activation of pancreatic enzymes, auto-digestion, and fibrosis, causing the replacement of the exocrine tissue generating PI, and the absence of residual exocrine tissue prevents the development of pancreatitis [8]. The pancreatic tissue replacement is an early event that likely begins in utero; in fact, all our pediatric subjects homozygous for the p.Phe508del mutation had PI since the first day of life. While we found all 33 cases of CP among CF subjects with other *CFTR* genotypes that more frequently include at least one mutation with residual function that exerts a dominant effect on the pancreatic status and warrants a residual activity of the exocrine tissue at the price of a higher risk to develop acute recurrent and/or CP [8]. However, such a simplistic view needs some comments. First, 9/33 (27.3%) CF subjects with CP had PI. This depends on the criterion used to define the pancreatic status that once was based on highly effective functional studies [12], while today it is mostly based on fecal elastase that lacks diagnostic sensitivity and specificity [13,14]. Moreover, even if most CF subjects with CP had at least one class 4–6 *CFTR* mutation, 8/33 (24.2%) CF subjects with CP had both class 1–3 *CFTR* mutations. Interestingly, 7/8 of these subjects had mutations in genes of the IPAT pathway that enhanced the risk of CP. In any case, the class definition of *CFTR* mutations is now questioned, since some *CFTR* mutations may bear to different classes, while for others rarer, the lack of functional studies renders it difficult to attribute the mutation to a specific class [15]. In addition, some *CFTR* mutations have been defined as high risk for pancreatitis and not for CF since they are associated with a normal chloride conductance (that prevents CF) but to an impaired bicarbonate conductance that may trigger pancreatitis [8]. This is the case of the p.Asp1152His and the p.Leu997Phe. Differently, in the present study, such mutations were found not only in CF subjects with CP but also in subjects with CF free from CP. On the other hand, a large study of our group demonstrated the highly variable phenotypic severity of the p.Asp1152His [16,17], and the same is true for the p.Leu997Phe. Thus, although the occurrence of CP to CF subjects is higher among CF subjects with PS, attention must be paid in all CF subjects, also considering that the treatment with modulators partially restores the integrity of the exocrine pancreas, enhancing the risk of developing pancreatitis [18,19]. Moreover, we suggest testing mutations that confer an increased risk of CP, at least in CF subjects with PI or who carry two severe mutations and develop CP. The presence of such mutations increases the risk of developing ductal adenocarcinoma [5], suggesting close monitoring of carriers and the counseling to extend molecular analysis to consanguineous.

The second subset of cases includes CFTR-RD, a group of conditions that do not fulfill all diagnostic criteria for CF diagnosis [20]. For a long time, CFTR-RD has been diagnosed occasionally, often in young adults, for symptoms, and once excluded, CF, they have not received treatments different from symptomatic approaches. In the last years, it emerged that such entities may have an evolutive trend, thus requiring monitoring, and the possibility of treatment with *CFTR* modulators [21] is discussed. Interestingly, a subset of CFTR-RD subjects appears with ARP or CP (and none of them had mutations in the genes of the IPAT pathway). No studies explored the long-term evolution of CFTR-RD subjects with pancreatitis so far and whether they have a higher risk for ductal adenocarcinoma, as it occurs for HP. Moreover, it is still unknown why subjects with CFTR-RD display so different monosymptomatic pictures.

Finally, we studied 136 subjects with iCP. About 26% had a family history according to the criteria to diagnose HP [5], but families still not diagnosed for pancreatitis, or subjects with CP not aware of the clinical conditions of all their families may mask a higher occurrence of family history. We found 66/136 (48.5%) cases with mutations in genes related to HP; such a figure compares with 65% of mutations found in 134 subjects with iCP in the United States [22] and 57% found in 715 Chinese subjects with iCP [23]. Among our 66 subjects carrying mutations, 4 had mutations in three different genes and 22 in two different genes, confirming that trans-heterozygosity enhances the risk for CP [7]. Moreover, we found 40 cases with a mutation in a single gene. The current view is that mutations in the *PRSS1* gene have a dominant inheritance, while mutations in other genes related to HP have a recessive inheritance [2,5]. While we speculate that also a single mutation in genes related to pancreatitis may represent a risk factor, this risk further increases with the number of genes mutated.

Among the mutated genes, the highest number of mutations involves *CFTR* (28 mutated alleles), and most of them (i.e., the p.Leu997Phe, the p.Ser1235Arg, the p.Asp1152His, the p.Arg74Gln, the p.Arg75Gln, and p.Arg170His) specifically impair bicarbonate permeability, representing a trigger for pancreatitis, but do not give rise to CF [9]. Differently, we found such mutations also in subjects with CF without CP and in subjects with CFTR-RD. Other mutations, mostly pathogenic [24], were found in the *PRSS1* gene (14 alleles). Two mutations of the *SPINK1* gene were found in both 4 single alleles. One of them, i.e., the p.Asn34Ser, is associated with a 12-fold increase of the risk to develop pancreatic adenocarcinoma [25], emphasizing the importance of offering genetic counseling to subjects carrying mutations and extending the analysis to consanguineous, in addition to planning monitoring to prevent or to early identify ductal adenocarcinoma. Other known mutations were found in the *CTRC* gene that encodes a protein that protects against the risk of pancreatitis degrading trypsinogen [26].

Moreover, we found mutations, mostly pathogenic, in 16 genes of the PSP in 31 alleles from subjects with CP, confirming that mutations in such genes might be related to the risk of CP [6,7]. We found 3 CP subjects carrying pathogenic mutations in the *CPA1* gene, strongly related to early-onset CP [27]. This gene encodes a protein that regulates the process of pancreatic proenzyme maturation and secretion. Similarly, three subjects had pathogenic mutations in the *TMPRSS15* gene that encodes enterokinase, the first enzyme involved in the activation of trypsinogen [28]. Moreover, we found a *TRPV1* and a *TRPV5* mutation in one of our subjects each. The possible role of such gene mutations in the pathogenesis of pancreatitis is yet to be defined. At the state of the art, it is known that TRPV1 channels are expressed by sensory neurons of the pancreas, and their activation triggers pancreatic inflammation [29].

Several genes encode for proteins related to the regulation of calcium flux into acinar cells, and the increased influx has an impact on the development of pancreatitis [30]. We found a mutation in the *DMBT1* gene and another in the *STIM1* gene, whose dysfunction is related to impaired regulation of calcium efflux [31]. Furthermore, we found 5 subjects with CP sharing the same *PIK3CG* gene mutation (i.e., the p.Ser442Tyr), previously found in 12 subjects with iCP [6]. The protein encoded by the *PIK3CG* gene plays multiple metabolic roles within acinar cells, among which the regulation of inflammatory response and mtROS production, which, once impaired, may trigger pancreatitis. Moreover, *PIK3CG* mutations are associated with metabolic reprogramming in pancreatic cancer [32]. Also, we found 4 mutations in the *SERPINA1* gene in 6 subjects with CP. It is known the increased risk to develop CP in subjects with liver steatosis and alpha1-antitrypsin deficiency so that serum A1AT was proposed as predictor of the risk to develop CP [33]. Other genes explored in our study encode for proteins related to the complex processes of acinar cell apoptosis, whose impairment may be responsible for CP [34]. This is the case of the *TNFRSF1A* gene, for which we found a CP patient with a pathogenic mutation. Also in the murine model, the deficiency of the tumor necrosis factor receptors generates a multiorgan inflammatory condition with pancreatic and hepatobiliary involvement [35]. Moreover, mutations in the carboxyl ester lipase gene (found in two cases of our cohort) are associated with both endocrine and, exocrine pancreas dysfunction called MODY8 [36] and more recently, with the risk of developing HP [37]. Finally, the impaired anion transport and the role of genes expressed in acinar cells gained interest in the pathogenesis of CP [38]. Among these genes we found mutations in *SLC26A3* (2 cases), *SLC4A4*, *ATP2C2,* and *DMBT1* (one case each).

The study has several limitations: (i) we cannot predict whether subjects with CFTR-RD would evolve to CF; (ii) most cases of pancreatitis were collected within the reference centers for CF, and this may represent a bias; (iii) the pancreatic status of CF subjects was assessed by fecal elastase that lacks diagnostic sensitivity and specificity; (iv) we have described the pancreatic status of subjects with CF before any treatment with modulators, not evaluating whether the treatment (that most of our subjects performed) changed the number of cases with PI or the number of cases who developed pancreatitis. However, the high number of subjects included in the study allows us to draw some conclusions.

## 4. Materials and Methods

### 4.1. Subjects

Table 4 describes the population of subjects included in the present study. In detail, we studied 377 subjects with CF, diagnosed by newborn screening (NBS) according to the criteria described [39], of which: (i) 263 adults, median age: 37 years, interquartile range (IQR): 31–48 years, 120 (45.6%) females, 70 (26.6%) homozygous for the p.Phe508del mutation, and 193 (73.4%) with other genotypes; (ii) 114 pediatric subjects, median age: 15 years, IQR: 14–17, 64 (56.1%) females, 45 (39.5%) homozygous for the p.Phe508del mutation, and 69 (60.5%) with other genotypes. Moreover, we studied 217 subjects with CFTR-RD diagnosed according to current standard criteria [20], of which: (i) 80 subjects with CP, median age: 29 years, IQR: 25–38 years, 41 (51.2%) females, none homozygous for the p.Phe508del mutation, and all of them with other *CFTR* genotypes; (ii) 45 subjects with DB, median age: 29 years, IQR: 23–35 years, 19 (42.2%) females, none homozygous for the p.Phe508del mutation, and all of them with other *CFTR* genotypes; (iii) 92 subjects with CBAVD, median age: 29 years, IQR: 24–32 years, all males, none homozygous for the p.Phe508del mutation and all of them with other *CFTR* genotypes. Finally, we studied 136 subjects that we grouped as CP, i.e., 44 (32.4%) with ARP and 92 with CP diagnosed according to the criteria described [40]. The median age was 27 years, IQR: 21–33 years, 60 (44.1%) females. In all subjects we excluded CF, CFTR-related disorders and all known causes of ARP/CP (i.e., gallstones, alcohol abuse, drugs, pancreatic malformation, autoimmunity), thus such subjects may be considered as iCP. Among such 136 subjects, 36 (26.5%) had family history, fulfilling the criteria for the diagnosis of HP [2].

### 4.2. Methods

From each subject included in the study, we recorded all clinical and anamnestic data. In the present paper we report the data of pancreatic status, pancreatitis, and sweat chloride (SC) before the eventual treatment with modulators. In particular, in all CF and CFTR-RD subjects we evaluated sweat chloride (SC) twice [41]; moreover, we performed *CFTR* mutation analysis by next-generation sequencing (NGS) carried out on the MiSeq 300SE platform (Illumina Inc., San Diego, CA, USA) [42] and tested genes encoding proteins potentially responsible for premature intrapancreatic activation of trypsinogen (IPAT genes) by NGS [6]. Subjects with CF were classified as pancreatic sufficient (PS) on the basis of at least two values of fecal elastase higher than 200 µg/g stool measured outside acute gastrointestinal diseases [43]. For subjects with CP, we tested by NGS either IPAT genes or genes encoding proteins related to the pancreatic secretion pathway (PSP) [6,7]. Data interpretation was performed using AmpliconSuite (SmartSeq SRL, Novara, Italy), version 3.7.0.

### 4.3. Statistical Analysis

Categorical data are reported as frequency (percentage) and compared by the chi-square test when expected frequencies are greater than 5. When expected frequencies were lower than 5, we applied Fisher’s exact test for 2 × 2 contingency tables and Fisher’s exact test with the Freeman-Halton extension for 3 × 2 contingency tables. Continuous data are reported as median (interquartile range, IQR) and compared by the Mann–Whitney U test (for two groups) and the Kruskal–Wallis test (for three groups). The Shapiro–Wilk test was applied to evaluate the normality of distributions. Statistical analyses were performed by SPSS (version 30, IBM SPSS Statistics, Armonk, NY, USA). We considered a significant *p* value < 0.01.

## 5. Conclusions

Subjects with CF may develop ARP and/or CP, and their number is increasing because the NBS allows for diagnosing a higher number of CF cases with PS that more frequently develop pancreatitis. However, CP may also appear in cases with PI and severe *CFTR* mutations (except for cases that are homozygous for the p.Phe508del mutation), and the risk is increased in subjects with mutations in PPGs. Thus, attention to the risk of CP must be paid to most subjects with CF testing for PGP genes and counselling all cases of CF with pancreatitis. Moreover, a subset of CFTR-RD appears with ARP/CP alone, and they are indistinguishable from CFTR-RD subjects that appear with other genotypes. Large multicenter studies are called to investigate why a subset of CFTR-RD appears with ARP/CP alone. Finally, we suggest a careful investigation of familiarity and the analysis of a large panel of PPG genes to better define the profile of risk for HP in all cases of idiopathic ARP/CP.

## Figures and Tables

**Table 1:** Pancreatic status of subjects with cystic fibrosis (CF).

Cohort	*N*	SC, mEq/L Median and IQR	PI *n* (%)	PS *n* (%)	Chronic Pancreatitis *n* (%)
Adult subjects					
p.Phe508del homozygous	70	101 (81–115) ^a^	69 (98.6%) ^b^	1 (1.4%) ^c^	0 (0.0%) ^c^
Other genotypes	193	85 (61–100)	124 (64.2%)	69 (35.8%)	21 (10.9%)
Pediatric subjects					
p.Phe508del homozygous	45	106 (91–116) ^a^	45 (100%) ^b^	0 (0.0%) ^c^	0 (0.0%) ^c^
Other genotypes	69	84 (69–109)	47 (68.1%)	22 (31.9%)	12 (17.4%)

SC: sweat chloride; PI: pancreatic insufficiency; PS: pancreatic sufficiency; IQR: interquartile range. ^a^ *p* < 0.001 (Mann-Whitney U test) as compared to CF subjects with other genotypes; ^b^ *p* < 0.005 (Chi-square test) as compared to CF subjects with other genotypes; ^c^ *p* < 0.005 (Fisher’s exact test) as compared to CF subjects with other genotypes.

**Table 2:** Features of subjects with cystic fibrosis (CF) and chronic pancreatitis (CP).

Group	*N*	Sweat Chloride, mEq/L Median (IQR)	PI *n* (%)	PS *n* (%)	1–3/1–3 Genotype *n* (%)	1–3/4–6 Genotype *n* (%)	Other Genotypes *n* (%)
Adult CF subjects with CP	21	68.0 (51.5–81.5)	8 (38.1%)	13 (61.9%)	6 (28.6%)	15 (71.4%)	0 (0.0%)
Adult CF subjects without CP	242	91.0 (64.0–106.0)	185 (76.4%)	57 (23.6%)	172 (71.1%)	58 (23.9%)	12 (5.0%)
*p*-value	—	0.006 ^a^	<0.001 ^b^	<0.001 ^b^	<0.001 ^b^	<0.001 ^b^	0.607 ^c^
Pediatric CF subjects with CP	12	40.0 (25.0–74.0)	1 (8.3%)	11 (91.7%)	2 (16.7%)	10 (83.3%)	0 (0.0%)
Pediatric CF subjects without CP	102	102.0 (80.0–116.0)	91 (89.2%)	11 (10.8%)	89 (87.3%)	13 (12.7%)	0 (0.0%)
*p*-value	—	<0.001 ^a^	<0.001 ^c^	<0.001 ^b^	<0.001 ^c^	<0.001 ^b^	—

Other genotype groups include 12 CF subjects with one or both unknown mutations. CP: chronic pancreatitis; PI: pancreatic insufficiency; PS: pancreatic sufficiency; IQR: interquartile range. (—) indicates that no numerical value is applicable for that specific cell. ^a^ Mann-Whitney U test; ^b^ Chi-square test; ^c^ Fisher’s exact test.

**Table 3:** Features of subjects with cystic fibrosis transmembrane regulator-related disorder (CFTR-RD).

Clinical Phenotype	*N*	Sweat Chloride, mEq/L Median (IQR)	PI *n* (%)	PS *n* (%)	1–3/1–3 ^a^ Genotype *n* (%)	1–3/4–6 ^b^ Genotype *n* (%)	Other Genotypes ^b^ *n* (%)
CFTR-RD with CP	80	36 (29–56)	0 (0.0%)	80 (100%)	1 (1.3%)	57 (71.3%)	22 (27.4%)
CFTR-RD with DB	45	39 (31–54)	0 (0.0%)	45 (100%)	0 (0.0%)	39 (86.7%)	6 (13.3%)
CFTR-RD with CBAVD	92	33 (29–56)	0 (0.0%)	92 (100%)	3 (3.3%)	82 (89.1%)	7 (7.6%)

The numbering 1–3 and 4–6 refers to the class of *CFTR* mutations. Other genotype groups include CF subjects with one or both unknown mutations. CP: chronic pancreatitis; DB: diffuse bronchiectasis; CBAVD: congenital bilateral absence of vas deferens; PI: pancreatic insufficiency; PS: pancreatic sufficiency; IQR: interquartile range. ^a^ *p* > 0.05, Fisher’s exact test with Freeman-Halton extension; ^b^ *p* > 0.05, chi-square test.

**Table 4:** Subjects included in the study.

Group	*N*	Males/Females	Age (Years), Median (IQR)	p.Phe508del Homozygous *n* (%)	Other Genotypes *n* (%)
Cystic Fibrosis (CF)					
Adult subjects	263	143/120	37 (31–48)	70 (26.6%)	193 (73.4%)
Pediatric subjects	114	50/64	15 (14–17)	45 (39.5%)	69 (60.5%)
CFTR-Related Disorder (CFTR-RD)					
CP	80	39/41	29 (25–38)	0 (0.0%)	80 (100%)
DB	45	26/19	29 (23–35)	0 (0.0%)	45 (100%)
CBAVD	92	92/0	29 (24–32)	0 (0.0%)	92 (100%)
Non-CF CP					
CP	136	76/60	27 (21–33)	—	—

CF: cystic fibrosis; CFTR-RD: cystic fibrosis transmembrane conductance regulator-related disorder; CP: chronic pancreatitis; DB: diffuse bronchiectasis; CBAVD: congenital bilateral absence of vas deferens; IQR: interquartile range. (—) indicates that no numerical value is applicable for that specific cell.

## Data Availability

The raw data supporting the conclusions of this article will be made available by the authors on request.

## Supplementary Materials

The following supporting information can be downloaded at: https://www.mdpi.com/article/10.3390/ijms27198620/s1.

## Abbreviations

The list of genes including abbreviations and OMIM numbers are reported in Supplemental Table S6.

The following abbreviations are used in this manuscript:

CP	chronic pancreatitis
HP	hereditary pancreatitis
ARP	acute recurrent pancreatitis
CF	cystic fibrosis
PPG	pancreatitis predisposing genes
IPAT	intrapancreatic activation of trypsinogen
PSP	pancreatic secretion pathway
PI	pancreatic insufficiency
CFTR	cystic fibrosis transmembrane conductance regulator
NBS	newborn screening
CFTR-RD	CFTR related disorder
DB	diffuse bronchiectasis
CBAVD	congenital bilateral absence of vas deferens
iCP	idiopathic chronic pancreatitis
SC	sweat chloride
NGS	next generation sequencing
IQR	interquartile range
PS	pancreatic sufficiency
