# Supplementary material for: Clinical and Genetic Interplay Between Chronic Pancreatitis, Cystic Fibrosis and CFTR-Related Disorders
Source: Int J Mol Sci. 2026 Sep 26;27(19):8620. doi: 10.3390/ijms27198620 (PMC13665086; doi:10.3390/ijms27198620)
Supplement: Supplementary file 1 [file ijms-27-08620-s001.zip › Table S1.pdf]

Table S1: *Cystic fibrosis transmembrane conductance regulator* (CFTR) genotype and mutations in genes related to intra-pancreatic activation of trypsinogen (IPAT) in 33 subjects with CF and chronic pancreatitis.

**CFTR Genotype**	**Mutation Class**	**N**	**PI / PS**	**IPAT Gene Mutations**
p.Phe508del / p.Asp1152His	1–3 / 4–6	7	3 / 4	—
p.Phe508del / p.Gly85Glu	1–3 / 4–6	2	2 / 0	—
p.Phe508del / p.Arg347Pro	1–3 / 4–6	2	0 / 2	—
p.Phe508del / c.2789+5G>A	1–3 / 4–6	1	0 / 1	—
p.Phe508del / c.3849+10kbC>T	1–3 / 4–6	1	0 / 1	—
p.Phe508del / p.Asp192Gly	1–3 / 1–3	1	1 / 0	*PRSS2*: p.Asn84Lys
p.Phe508del / p.Asp614Gly	1–3 / 1–3	1	1 / 0	*CTRC*: p.Gly217Ser
p.Phe508del / p.Ile1027Thr	1–3 / 1–3	1	1 / 0	*CTRC*: p.Lys172Glu
p.Phe508del / p.Gly27Glu	1–3 / 1–3	1	0 / 1	—
p.Phe508del / p.Lys1461Serfs*7	1–3 / 1–3	1	0 / 1	*PRSS1*: p.Leu104Pro
p.Phe508del / p.Ser549Asn	1–3 / 4–6	1	0 / 1	*CTRC*: p.Gly217Ser
p.Asn1303Lys / c.2789+5G>A	1–3 / 4–6	1	0 / 1	—
p.Asn1303Lys / p.Arg74Trp	1–3 / 4–6	1	0 / 1	—
p.Asn1303Lys / p.Pro205Ser	1–3 / 1–3	1	0 / 1	*PRSS2*: p.Asn84Lys
p.Asn1303Lys / p.Leu997Phe	1–3 / 4–6	1	0 / 1	—
p.Gly542* / c.2789+5G>A	1–3 / 4–6	1	0 / 1	—
p.Gly542* / p.Glu1418Argfs*14	1–3 / 1–3	1	0 / 1	*PRSS1*: p.Cys139Phe
p.Gly542* / p.Leu997Phe	1–3 / 4–6	1	0 / 1	—

CFTR Genotype	Mutation Class	N	PI / PS	IPAT Gene Mutations
p.Gly1069Arg / p.Phe1052Val	1–3 / 1–3	1	0 / 1	*PRSS2*: p.Asn84Lys
p.Gly85Glu / [p.Ile1023_Val1024del; p.Ile148Thr]	1–3 / 4–6	1	1 / 0	—
p.Pro5Leu / p.Asp1152His	1–3 / 4–6	1	0 / 1	—
p.Arg117His / p.Arg334Trp	1–3 / 4–6	1	0 / 1	—
p.Asp1152His / p.Tyr849*	1–3 / 4–6	1	0 / 1	—
p.Glu1433Gly/ p.Leu997Phe	1–3 / 4–6	1	0 / 1	—
p.Gly1244Glu / c.3849+10kbC>T	1–3 / 4–6	1	0 / 1	—

PI: pancreatic insufficiency; PS: pancreatic sufficiency. (—) indicates that no IPAT Gene Mutations is applicable for that specific cell
