# Supplementary material for: Clinical and Genetic Interplay Between Chronic Pancreatitis, Cystic Fibrosis and CFTR-Related Disorders
Source: Int J Mol Sci. 2026 Sep 26;27(19):8620. doi: 10.3390/ijms27198620 (PMC13665086; doi:10.3390/ijms27198620)
Supplement: Supplementary file 1 [file ijms-27-08620-s001.zip › Table S2.pdf]

Table S2: Number and (%) of *cystic fibrosis transmembrane conductance regulator* (CFTR) mutations found in 33 cystic fibrosis (CF) subjects (66 alleles) with chronic pancreatitis (CP) compared to 344 CF subjects (688 alleles) without CP.

**CFTR Mutation**	**CF with CP (*n* = 66 alleles)**	**CF without CP (*n* = 688 alleles)**	***p*-value**
p.Phe508del	19 (28.8%)	413 (60.0%)	< 0.001 [a]
p.Asp1152His	9 (13.6%)	6 (0.9%)	< 0.001 [a]
p.Asn1303Lys	4 (6.0%)	31 (4.5%)	ns [b]
p.Gly542*	3 (4.5%)	19 (2.8%)	ns [b]
c.2789+5G>A	3 (4.5%)	7 (1.0%)	0.049 [b]
p.Leu997Phe	3 (4.5%)	2 (0.3%)	0.006 [b]
p.Gly85Glu	3 (4.5%)	4 (0.6%)	0.017 [b]
c.3849+10kbC>T	2 (3.0%)	5 (0.7%)	ns [b]
p.Arg347Pro	2 (3.0%)	3 (0.4%)	ns [b]
p.Asp192Gly	1 (1.5%)	0 (0.0%)	ns [b]
p.Asp614Gly	1 (1.5%)	0 (0.0%)	ns [b]
p.Ile1027Thr	1 (1.5%)	1 (0.1%)	ns [b]
p.Gly27Glu	1 (1.5%)	0 (0.0%)	ns [b]
p.Lys1461Serfs*7	1 (1.5%)	0 (0.0%)	ns [b]
p.Ser549Asn	1 (1.5%)	2 (0.3%)	ns [b]
p.Arg74Trp	1 (1.5%)	0 (0.0%)	ns [b]
p.Pro205Ser	1 (1.5%)	0 (0.0%)	ns [b]
p.Glu1418Argfs*14	1 (1.5%)	0 (0.0%)	ns [b]
p.Gly1069Arg	1 (1.5%)	1 (0.1%)	ns [b]

**CFTR Mutation**	**CF with CP (*n* = 66 alleles)**	**CF without CP (*n* = 688 alleles)**	***p*-value**
p.Phe1052Val	1 (1.5%)	2 (0.3%)	ns [b]
[p.Ile1023_Val1024del;p.Ile148Thr]	1 (1.5%)	0 (0.0%)	ns [b]
p.Pro5Leu	1 (1.5%)	1 (0.1%)	ns [b]
p.Arg117His	1 (1.5%)	1 (0.1%)	ns [b]
p.Arg334Trp	1 (1.5%)	0 (0.0%)	ns [b]
p.Tyr849*	1 (1.5%)	2 (0.3%)	ns [b]
p.Glu1433Gly	1 (1.5%)	2 (0.3%)	ns [b]
p.Gly1244Glu	1 (1.5%)	5 (0.7%)	ns [b]

The Table lists only CFTR mutations found in at least one allele from subjects with CP.
CF: cystic fibrosis; CFTR: *cystic fibrosis transmembrane conductance regulator*; CP: chronic pancreatitis; ns: not significant. [a] Chi-square test; [b] Fisher's exact test.
