# Supplementary material for: Clinical and Genetic Interplay Between Chronic Pancreatitis, Cystic Fibrosis and CFTR-Related Disorders
Source: Int J Mol Sci. 2026 Sep 26;27(19):8620. doi: 10.3390/ijms27198620 (PMC13665086; doi:10.3390/ijms27198620)
Supplement: Supplementary file 1 [file ijms-27-08620-s001.zip › Table S3.pdf]

Table S3: Number and (%) of mutations in genes related to intra-pancreatic activation of trypsinogen (IPAT) in cystic fibrosis (CF) subjects with and without chronic pancreatitis (CP).

**IPAT Gene Mutations**	**CF Subjects with CP (n = 33)**	**CF Subjects without CP (n = 344)**	***p*-value [a]**
PRSS1: p.Leu104Pro	1 (3.0%)	2 (0.6%)	ns
PRSS1: p.Cys139Phe	1 (3.0%)	1 (0.3%)	0.038
PRSS2: p.Asn84Lys	3 (9.1%)	0 (0.0%)	< 0.001
CTRC: p.Gly217Ser	1 (3.0%)	0 (0.0%)	0.001
CTRC: p.Lys172Glu	2 (6.1%)	1 (0.3%)	< 0.001
SPINK1: p.Pro55Ser	0 (0.0%)	1 (0.3%)	ns
**Total**	**8 (24.2%)**	**5 (1.5%)**	**< 0.001**

CF: cystic fibrosis; CP: chronic pancreatitis; IPAT: intra-pancreatic activation of trypsinogen; ns: not significant.
[a] Chi-square test.
