# Supplementary material for: Clinical and Genetic Interplay Between Chronic Pancreatitis, Cystic Fibrosis and CFTR-Related Disorders
Source: Int J Mol Sci. 2026 Sep 26;27(19):8620. doi: 10.3390/ijms27198620 (PMC13665086; doi:10.3390/ijms27198620)
Supplement: Supplementary file 1 [file ijms-27-08620-s001.zip › Table S4.pdf]

Table S4: Cystic fibrosis transmembrane conductance regulator (CFTR) mutations, number and (%), found in subjects with CFTR-related disorders (CFTR-RD) with chronic pancreatitis (CP), diffuse bronchiectasis (DB), and congenital bilateral absence of vas deferens (CBAVD).

**Mutation**	**CP (160 alleles)**	**DB (90 alleles)**	**CBAVD (184 alleles)**	***p*-value**
p.Phe508del	33 (20.6%)	20 (22.2%)	40 (21.7%)	ns [a]
p.Asp1152His	22 (13.8%)	6 (6.7%)	9 (4.9%)	0.010 [a]
c.5T-TG12	19 (11.9%)	12 (13.3%)	24 (13.0%)	ns [a]
p.Leu997Phe	10 (6.3%)	3 (3.3%)	4 (2.2%)	ns [b]
p.Gly542*	9 (5.6%)	6 (6.7%)	10 (5.4%)	ns [a]
p.Arg347Pro	7 (4.4%)	4 (4.4%)	9 (4.9%)	ns [b]
p.Gly85Glu	8 (5.0%)	4 (4.4%)	5 (2.7%)	ns [b]
p.Arg553*	5 (3.1%)	3 (3.3%)	5 (2.7%)	ns [b]
[p.Arg74Trp;p.Asp1270Asn]	4 (2.5%)	1 (1.1%)	1 (0.5%)	ns [b]
c.711+1G>T	4 (2.5%)	0 (0.0%)	3 (1.6%)	ns [b]
p.Ser1235Arg	4 (2.5%)	0 (0.0%)	2 (1.1%)	ns [b]
p.Arg1158*	3 (1.9%)	0 (0.0%)	3 (1.6%)	ns [b]
p.Arg334Trp	3 (1.9%)	2 (2.2%)	5 (2.7%)	ns [b]
p.Leu1065Pro	3 (1.9%)	1 (1.1%)	1 (0.5%)	ns [b]
p.Asp110His	2 (1.3%)	1 (1.1%)	2 (1.1%)	ns [b]
p.Ser1297Phefs*5	2 (1.3%)	1 (1.1%)	5 (2.7%)	ns [b]
p.Asp614Gly	2 (1.3%)	0 (0.0%)	1 (0.5%)	ns [b]
p.Arg117His	2 (1.3%)	2 (2.2%)	5 (2.7%)	ns [b]
p.Leu145His	1 (0.6%)	0 (0.0%)	0 (0.0%)	ns [b]

Mutation	CP<br>(160 alleles)	DB<br>(90 alleles)	CBAVD<br>(184 alleles)	*p*-value
p.Arg1162Leu	1 (0.6%)	2 (2.2%)	1 (0.5%)	ns [b]
p.Asn1303Lys	1 (0.6%)	2 (2.2%)	6 (3.3%)	ns [b]
p.Lys684Serfs*38	1 (0.6%)	2 (2.2%)	5 (2.7%)	ns [b]
p. Pro499Ala	1 (0.6%)	0 (0.0%)	1 (0.5%)	ns [b]
c.2789+5G>A	0 (0.0%)	1 (1.1%)	3 (1.6%)	ns [b]
c.3849+10kbC>T	0 (0.0%)	2 (2.2%)	3 (1.6%)	ns [b]
Unknown	13 (8.1%)	15 (16.7%)	31 (16.8%)	-

CBAVD: congenital bilateral absence of vas deferens; CFTR: cystic fibrosis transmembrane conductance regulator; CFTR-RD: CFTR-related disorders; CP: chronic pancreatitis; DB: diffuse bronchiectasis. (—) indicates that no numerical value is applicable for that specific cell [a] Chi-square test. [b] Fisher's exact test with Freeman-Halton extension.
