# Supplementary material for: Clinical and Genetic Interplay Between Chronic Pancreatitis, Cystic Fibrosis and CFTR-Related Disorders
Source: Int J Mol Sci. 2026 Sep 26;27(19):8620. doi: 10.3390/ijms27198620 (PMC13665086; doi:10.3390/ijms27198620)
Supplement: Supplementary file 1 [file ijms-27-08620-s001.zip › Table S5.pdf]

Table S5: Genotypes of genes related to intra-pancreatic activation of trypsinogen (IPAT, underlined) and genes related to pancreatic secretion pathways (PSP genes) in 136 patients with chronic pancreatitis. Table reports also the cases of familiarity.

Genotypes	Familiarity	Number of cases
CFTR: p.Gln39His; SERPINA1: p.Glu288Val; TMPRSS15: p.Ser712*		1
CFTR: p.Ser1297Phefs*5; PRSS1: p.Gly177*; SERPINA1: p.Gly288Val	1/1	1
PRSS1: p.Ala16Val; SERPINA 1: p.Gly366Leu; KMT2D: p.Gly1713Arg	1/1	1
SPINK1 p.Asn34Ser; SERPINA1: p.Glu288Val; PNLIP: p.Gln323Pro	1/1	1
CFTR: p.Leu997Phe; CFTR: p.Asp1152His	1/1	1
CFTR: p.Arg75Gln; PRSS1: p.Asn29Ile		1
CFTR: c.5T-TG12; PRSS2: c.200+1G>T	1/1	1
CFTR: p.Ser1235Arg; SERPINA1: p.Asp280Val		1
CFTR: p.Lys68Glu ; CPA1: p.Arg240Tyr		1
CFTR: p.Leu997Phe; CTRC: p.Leu172Gly		1
CFTR: p.Arg74Gln; SPINK1: p.Asn34Ser		1
PRSS1: p.Asn29Ile; CTRC: p.Thr230Ile		1
PRSS1: p.Cys171*; PICK3CG: p.Ser442Tyr	1/1	1
PRSS1: p.Asn29Ile; CTRC: p.Lys172Glu		1
PRSS1: p.Arg122His; PRSS2: p.Asp153His	1/1	1
PRSS1: p.Gly177Val/p.Gly177Val	1/1	1
PRSS1: p.Gly177Val; SERPINA1: p.Pro393Ser		1
PRSS1: p.Ala16Val; SLC4A4: p.Ile326Val		1
PRSS1: p.Cys139Phe; SPINK1: p.Asn34Ser		1
SPINK1: p.Pro55Ser; ATP2C2: p.Gly231Trp	1/1	1
SPINK1: p.Pro55Ser; TNFRSF1A: p.Arg312Lys		1
SPINK1: p.Pro55Ser; TRPV5: p.Ala563Thr		1
CTRC: p.Val235Ile; TRPV1: p. Asp746=		1
KRT8: p.Val380Ile; STIM1: p.ArgR749His		1
TMPRSS15: p.Asp620His; SLC26A3: p.Arg571His		1
PICK3CG: p.Ser442Tyr; DMBT1: p.Arg806Trp		1
CFTR: p.Leu997Phe	2/5	5
CFTR: p.Ser1235Arg		2
CFTR: c.5T-TG12		1
CFTR: p.Arg75Gln		1
CFTR: p.Asp614Gly		1
CFTR: p.Arg74Gln		1
CFTR: p.Arg170His		2
CFTR: p.Ile807Met		1
CFTR: p.Ser1235Arg		1
CFTR: p.Tyr1381His		1
CFTR: p.Tyr84His		1
CFTR: p.Val1397Glu		1
PRSS1: p.Arg122His	1/2	2
PRSS1: p.Ala16Val		1
PRSS2: p.Asp153His		1

SPINK1: p.Pro45Ser		3
SPINK1: p.Asn34Ser		2
CTRC: p.Arg119Cys		1
CTRC: p.Val235Ile		1
CTSB: p.Ala327Ser		1
CEL: p.Thr388Ser		1
CEL: p.Val593Argfs*6		1
CPA1: p.Asn256Ser		1
CPA1: p.Gly277Ser		1
IL21: p.Ile18Val		1
PICK3CG: p.Ser442Tyr		3
SLC26A3: p.Lys723Asn		1
TMPRSS15: p.L935Serfs*6		1
Absence of mutations	20/70	70
