# Supplementary material for: Clinical and Genetic Interplay Between Chronic Pancreatitis, Cystic Fibrosis and CFTR-Related Disorders
Source: Int J Mol Sci. 2026 Sep 26;27(19):8620. doi: 10.3390/ijms27198620 (PMC13665086; doi:10.3390/ijms27198620)
Supplement: Supplementary file 1 [file ijms-27-08620-s001.zip › Table S6.pdf]

Table S6. Mutations in genes related to intra-pancreatic activation of trypsinogen (IPAT) and genes related to pancreatic secretion pathways (PSP genes) in 136 patients (272 alleles) with chronic pancreatitis.

Gene	OMIM	Mutation	Pathway	Mutated alleles (*n*)
CFTR (cystic fibrosis transmembrane regulator)	602421	p.Leu997Phe	IPAT	8
		p.Ser1235Arg	IPAT	3
		p.Asp1152His	IPAT	3
		5T-TG12	IPAT	2
		p.Arg74Gln	IPAT	2
		p.Arg75Gln	IPAT	2
		p.Arg170His	IPAT	2
		p.Gln39His	IPAT	1
		p.Lys68Glu	IPAT	1
		p.Tyr84His	IPAT	1
		p.Val397Leu	IPAT	1
		p.Ser1297Phefs*5	IPAT	1
		p.Thr1381His p.Tyr1381His	IPAT	1
PRSS1 (serine protease 1)	276000	p.Arg122His	IPAT	6
		p.Ala16Val	IPAT	3
		p.Asn29Ile	IPAT	3
		p.Gly177Val	IPAT	3
		p.Cys139Phe	IPAT	1
		p.Cys171*	IPAT	1
SPINK1	167790	p.Asn34Ser	IPAT	4
		p.Pro55Ser	IPAT	4
PRSS2	601564	p.Asp153His	IPAT	2
		c.200+1G>T	IPAT	1
CTRC	601405	p.Leu172Gly	IPAT	2
		p.Val235Ile	IPAT	2
		p.Arg119Cys	IPAT	1
		p.Thr230Ile	IPAT	1
KRT8	148060	p.Val380Ile	IPAT	1
CTSB	116810	p.Ala327Ser	IPAT	1
PIK3CG	601232	p.Ser442Tyr	PSP	5
SERPINA1	107400	p.Glu288Val	PSP	3
		p.Asp280Val	PSP	1
		p.Gly366Leu	PSP	1
		p.Pro393Ser	PSP	1
TMPRSS15	606635	p.Asp620His	PSP	1
		p.Ser712*	PSP	1
		p.L935Serfs*6	PSP	1
CPA1	114850	p.Arg240Tyr	PSP	1
		p.Asn256Ser	PSP	1

		p.Gly277Ser	PSP	1
CEL	114840	p.Thr388Ser	PSP	1
		p.Val593Argfs*6	PSP	1
SLC26A3	126650	p.Arg571His	PSP	1
		p.Lys723Asn	PSP	1
ATP2C2	613082	p.Gly231Trp	PSP	1
DMBT1	601969	p.Arg806Trp	PSP	1
KMT2D	602113	p.Gly1713Arg	PSP	1
IL21	605384	p.Ile18Val	PSP	1
PNLIP	246600	p.Gln323Pro	PSP	1
SLC4A4	603345	p.Ile326Val	PSP	1
STIM1	605921	p.Arg749His	PSP	1
TNFRSF1A	191190	p.Arg312Lys	PSP	1
TRPV1	602076	p.Asp746=	PSP	1
TRPV5	606679	p.Ala563Thr	PSP	1
